# Electron self-exchange activation parameters of diethyl sulfide and tetrahydrothiophene

**DOI:** 10.3762/bjoc.9.164

**Published:** 2013-07-19

**Authors:** Martin Goez, Martin Vogtherr

**Affiliations:** 1 Institut für Chemie, Martin–Luther-Universität Halle–Wittenberg, Kurt–Mothes-Str. 2, 06120 Halle/Saale, Germany; 2 Merck KGaA, NMR Spectroscopy, Frankfurter Straße 250, 64293 Darmstadt, Germany

**Keywords:** CIDNP, electron transfer, free radicals, kinetics, photochemistry, pyrylium salts, self-exchange, sulfides

## Abstract

Electron transfer between the title compounds and their radical cations, which were generated by photoinduced electron transfer from the sulfides to excited 2,4,6-triphenylpyrylium cations, was investigated by time-resolved measurements of chemically induced dynamic nuclear polarization (CIDNP) in acetonitrile. The strongly negative activation entropies provide evidence for an associative–dissociative electron exchange involving dimeric radical cations. Despite this mechanistic complication, the free energies of activation were found to be well reproduced by the Marcus theory of electron transfer, with the activation barrier still dominated by solvent reorganization.

## Introduction

Single-electron transfer is probably the simplest chemical process of an organic molecule, because usually no full bonds are broken or formed. For this reaction type, the relationship between its thermodynamic driving force and its rate is well understood [1–2]; it only depends on a single parameter for each reagent involved, namely, the activation barrier 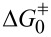 of its self-exchange, e.g.,

[1]
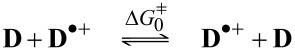


Observing these key reactions is complicated by the fact that they involve no change of the sample composition. To make the two sides of the reaction equation distinguishable, it is mandatory to label the reagents; then one can measure the transfer rate of the labels to the other species. For fast reactions, nuclear or electron spins are the only labels that can be applied without disturbing the energetics and kinetics. NMR exchange spectroscopy [3] or EPR line broadening experiments [4] employ radiofrequency pulse schemes to generate a suitable magnetization or coherence for this purpose; however, as all such self-exchanges by necessity involve radical species, an ”automatic” labeling with nonequilibrium magnetizations is also feasible by utilizing the CIDNP [5–10] effect (chemically induced dynamic nuclear polarization).

CIDNP arises through a complex interplay of nuclear-spin-selective intersystem crossing and electron-spin-selective reactivity. Its key intermediates are radical pairs 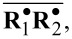 i.e., two radicals possessing a correlation of their electron spins, which originates from the electron spin multiplicity of their precursor. The two radicals of each spin-correlated pair undergo diffusive separations and may reencounter at a later time. During their excursions, the differential precession of their electron spins causes intersystem crossing with a rate that depends on the spin states of their nuclei through the hyperfine interactions; upon reencounter, a chemical reaction serves to distinguish between singlet and triplet pairs (usually, a geminate reaction, i.e., a reaction of the two radicals with each other, is only feasible in the singlet state, whereas the radicals of the triplet pairs escape and ultimately react with other molecules in the sample, i.e., individually). In conjunction, these two processes sort the nuclear spins within the life of the pairs (typically, less than 1 ns) such that polarizations, i.e., nonequilibrium magnetizations, of exactly equal magnitudes but opposite signs are generated in the products from the singlet and triplet exit channels. These experiments are normally carried out in the magnet of an NMR spectrometer, so the polarizations in the diamagnetic products can be directly sampled by a radiofrequency pulse and manifest themselves as anomalous line intensities in the NMR spectrum.

Given an appropriate acceptor/donor combination **A**/**D**, photoinduced electron transfer [11] is an expedient route to radical pairs 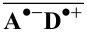 by laser flash photolysis. Without further action on the part of the experimenter, the radical pair mechanism then creates opposite polarizations in the geminate products, i.e., in the starting materials **A** and **D** regenerated by reverse electron transfer, and in the escaping free radicals **A^•^**^−^ and **D^•^**^+^, in other words, labels all four species with their respective polarizations, e.g., **D_↑_** and 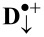. As was first recognized by Closs [12], the self-exchange leads to a gradual cancelation of the opposite polarizations in each substrate and its corresponding radical ion, e.g.,

[2]
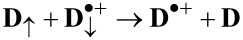


on a timescale of 1...100 microseconds for suitably chosen substrate concentrations. A radiofrequency pulse (typical duration: microseconds for protons) applied at a certain point of time after the laser flash converts the polarizations present in **D** at that precise moment into observable coherences and isolates them from the further cancelation, which only operates for magnetizations. Hence, a series of such time-resolved CIDNP experiments [13–16] with different delays between laser flash and observation pulse provides a direct way of measuring the self-exchange rates [17–20]. Practically all published studies of electron self-exchange – apart from indirect determinations based on the Marcus cross relationship [2] – have been carried out on aromatic or olefinic substrates. All known aliphatic radical cations are heteroatom-centered, which considerably increases the mechanistic intricacies. The main complication arises from fast deprotonation of **D^•^**^+^ at the α carbon to give a neutral radical 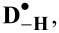 which can occur at the radical-pair stage in a direct reaction (base, **A^•^**^−^) or even at any stage of the reaction by a relayed proton transfer (relay base, **D**) [21–23] and turns the gross reaction 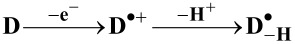 into a hydrogen abstraction. In a previous report on two aliphatic amines [20], we have used time-resolved CIDNP experiments to distinguish between the ensuing electron and hydrogen self-exchanges of **D^•^**^+^ and 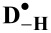. In the present work, we investigate two structurally similar aliphatic sulfides, diethyl sulfide **DES** and tetrahydrothiophene **THTP**, for which we have barred the direct deprotonation at C_α_ by the choice of a suitable sensitizer; for these substrates, a relayed deprotonation is also impossible because they are not sufficiently basic; however, **D^•^**^+^ can stabilize by dimer formation with surplus **D** to give 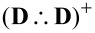 [24]. Except for an order-of-magnitude estimate of the exchange rate from EPR line broadening [25] and a brief remark in an earlier communication by us [26], this is the first report on direct measurements of the electron self-exchange of such sulfur-centered aliphatic radical cations. As we will show, the activation parameters provide evidence that the observed exchange indeed takes place between dimeric radical cations and **D**.

## Results and Discussion

On the AM1 level, the heats of formation of **D** and 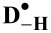 are −92 kJ/mol and −14 kJ/mol for diethyl sulfide **DES** as opposed to −69 kJ/mol and +85 kJ/mol for triethylamine, the quencher used in our earlier work [21–23], so for any sensitizer the radical pair containing 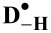 lies about 75 kJ/mol higher above the reactants in the case of the amine. From the oxidation potentials in the literature (**DES**, 1.65 V [27]; triethylamine, 0.96 V [21]; both in acetonitrile versus SCE) the opposite order, even with a numerically similar difference, is calculated for the radical pair with **D^•^**^+^, although observations [28–29] that for bifunctional donors comprising both an amine and a sulfide moiety photoinduced electron transfer occurs exclusively from **S**, not **N**, cast some doubt on whether these reported potentials are the true equilibrium potentials for these irreversible redox systems. In any case, a sensitizer that is a potent electron acceptor but has no hydrogen-abstracting power is mandatory to shift the energetics so as to stop the photoreaction after the stage of the electron transfer.

Two other demands on the sensitizer are imposed by the necessity of maximizing the CIDNP effects, because intrinsically low enhancement factors are expected in consequence [6] of the very high *g* values of sulfur-centered radical cations. First, to suppress instant electron return and thereby maximize the number of radical pairs available for generating polarizations, a triplet sensitizer should be used [11]; the long life of a triplet excited state is also the only way to meet the conflicting requirements of bringing the self-exchange rates into the observable kinetic range by correspondingly low donor concentrations while still retaining a sufficient amount of quenching. Second, that sensitizer should be positively charged, because this removes the Coulombic attraction between the radicals of the pair, so they can easily separate to a distance where spin mixing becomes effective [30].

All these conditions are fulfilled by the 2,4,6-triphenylpyrylium cation **TTP**^+^. Compared to typical carbonyl sensitizers such as benzophenone or xanthone, its reduction potential (−0.29 V in acetonitrile versus SCE [31]) is more favorable by more than 2 V, yet its triplet energy (222 kJ/mol [32]) is only lower by less than 90 kJ/mol. Its first excited singlet state has a lifetime of 2.9 ns [32], so reacts only marginally with the millimolar sulfide concentrations used in our experiments, but its triplet state (intrinsic intersystem-crossing efficiency, 0.48 [32], with evidence for a noticeable increase in the presence of **DES** by an intermolecular heavy-atom effect [33]) is quenched [34] quantitatively (lifetime, 10 μs [32]; diffusion-controlled electron transfer from **DES** [35]) under our experimental conditions. Protonation of the pyranyl radical **TPP**^•^ is unknown.

**Figure 1:**
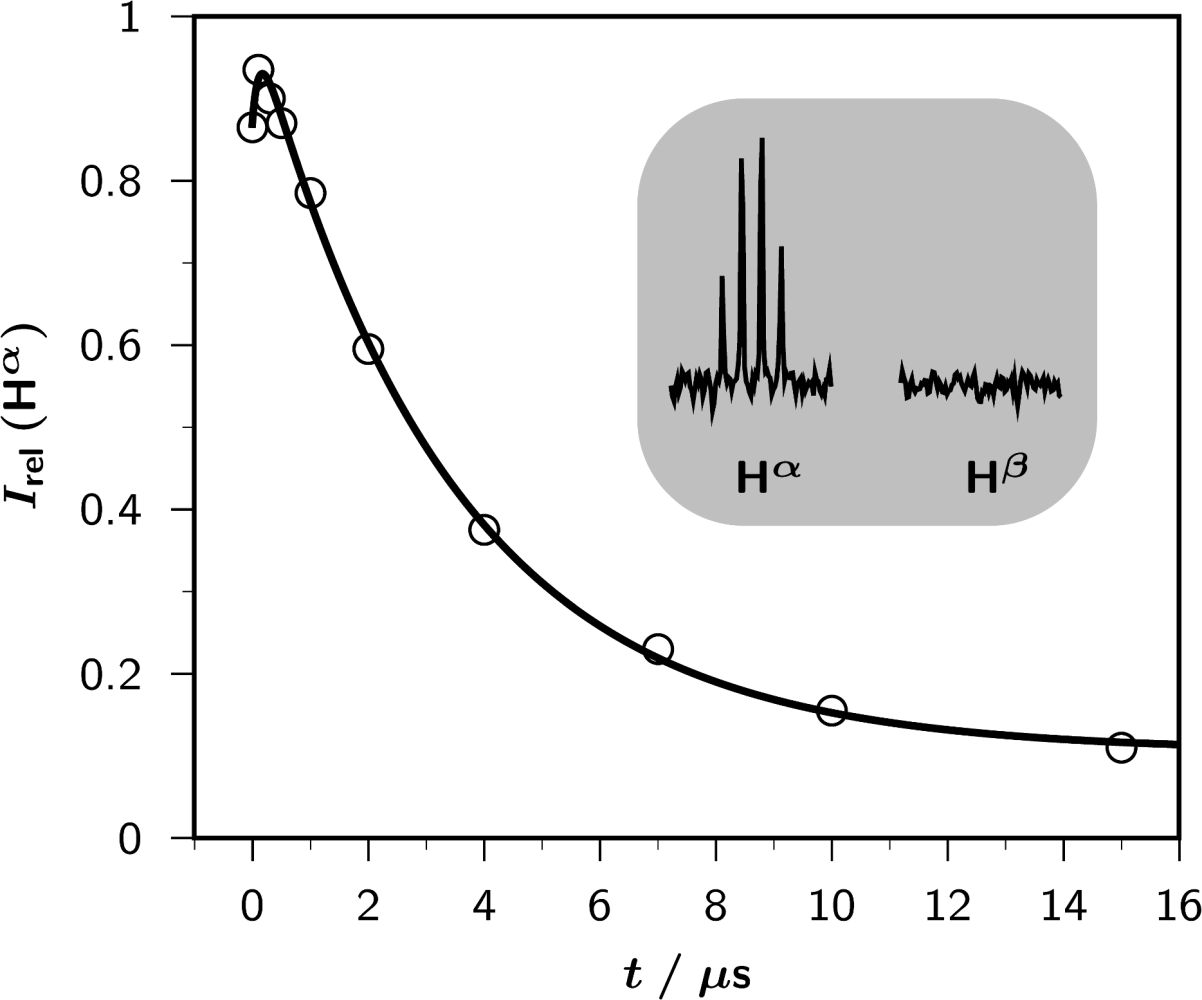
Time-resolved CIDNP measurements on a sample of 5 × 10^−3^ M **DES** in acetonitrile-*d*_3_ sensitized by 2 × 10^−4^ M **TPP**^+^; temperature, 278 K.The main plot shows the relative CIDNP intensities (integrals) *I*_rel_(**H**^α^) of the α protons of the starting sulfide as functions of the delay *t* between the laser flash (wavelength, 308 nm) and the NMR observation pulse (width, 1 μs; tip angle, 22.5°).The fit function is given by Equation 3, in conjunction with Equation 4 and Equation 5. Best-fit kinetic parameters: *k*_0_, 8.80 × 10^6^ s^−1^, corresponding to a quenching rate constant of 1.8 × 10^9^ M^−1^ s^−1^; *k*_ex_, 5.37 × 10^7^ M^−1^ s^−1^; *T*_1_, 36.5 μs.The inset displays the actual CIDNP signals of the α and β protons of **DES** (**H**^α^, 2.51 ppm; **H**^β^, 1.20 ppm) at a delay of 0 μs. Further explanation, see text.

The CIDNP effects observed in the photoreaction of **DES** sensitized by **TPP^+^** in acetonitrile are typical [12] for a self-exchange: Only the starting materials exhibit CIDNP, i.e., no other spin-polarized products are formed; hence, the radicals must ultimately stabilize by reverse electron transfer. In spectra with continuous illumination, the polarizations are only weak whereas time-resolved experiments (Figure 1) reveal that they are much stronger initially but quickly decay to a low residual level; this cancelation is clear evidence for an exchange between the sulfide-derived radical and its parent compound.

Which sulfide-derived intermediate is the source of CIDNP can be concluded from the the polarization pattern, i.e., from the relative polarization intensities of the different protons; ethyl groups are very convenient for this type of analysis [36]. In an ethyl-substituted heteroatom-centered radical cation, such as **DES^•^**^+^, only the α protons experience an appreciable hyperfine coupling, so only these protons can acquire polarizations. In contrast, for an α (heteroatom) substituted ethyl radical, e.g., 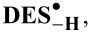 both the α and the β protons possess large hyperfine coupling constants, which are negative and positive, respectively. This would translate into a very different polarization pattern, namely, CIDNP signals of similar magnitude but opposite sign for those two types of protons. As the inset of Figure 1 reveals, only the α protons are polarized in our system, which is thus incontrovertible evidence for the intermediacy of a radical pair containing **DES^•^**^+^.

A more complete analysis can be given with Kaptein’s rule [37] for the polarization phase Γ*_i_* of nucleus *i* in a reaction product (Γ*_i_* = +1, absorption; Γ*_i_* = −1, emission),

[6]
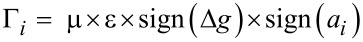


In Equation 6, the parameters μ and ε characterize the reaction pathway (μ, precursor multiplicity leading to the radical pair: μ = +1, triplet, μ = −1, singlet; ε, radical-pair multiplicity from which the product is formed: ε = +1, singlet, ε = −1, triplet), and Δ*g* and *a_i_* are the pertinent magnetic properties of the radical pair (Δ*g*, *g*-value difference of the two radicals, with the one containing nucleus *i* taken first; *a_i_*, hyperfine coupling constant of that nucleus in the radical). Together with the fact that the absolute CIDNP intensity of nucleus *i* is approximately proportional to *a_i_* [7], this sign rule forms the basis for identifying a paramagnetic intermediate through the resulting polarization pattern.

The *g* value of **DES^•^**^+^, regardless of whether it is present as a monomeric radical cation (*g* = 2.017 [38]) or in its dimeric form (*g* = 2.011 [25]), is much larger than that of the pyranyl radical **TPP^•^** (*g* = 2.0031 [39]), so Δ*g* must be positive for the sulfide protons; as stated above, the hyperfine coupling constant of **H**^α^ is also positive. For the radical pair, reverse electron transfer is only possible in its singlet state (ε = +1), because the triplet state of **DES** (281 kJ/mol [40]) lies even higher in energy than the initial excited singlet state of **TPP^+^** (272 kJ/mol [32]). The absorptive polarization for **H**^α^ thus confirms the precursor multiplicity expected on the basis of the thermodynamic and photophysical data, i.e., triplet (μ = +1).

The absence of polarizations in the sensitizer is easily rationalized by the low hyperfine coupling constants in its radical **TPP^•^** (between 2.5 G and 0.4 G [39], as opposed to 18–20 G for **H**^α^ in the monomeric [38] and 6.8 G in the dimeric [25] radical cation of the sulfide) in conjunction with its complicated spectral habit, which causes the polarizations to be distributed among many resonances, with concomitant decrease in intensity of the individual peaks.

For tetrahydrothiophene **THTP**, the same polarization pattern (polarizations for **H**^α^ only, i.e., CIDNP originating from radical pairs 
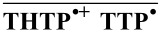
) and kinetic behavior of the polarizations was found, which is not surprising given the very similar thermodynamics (ionization potentials of **DES** and **THTP**, 8.42 eV and 8.62 eV, respectively [41]) and magnetic parameters (monomeric **THTP^•^**^+^ [38], *g* = 2.014, *a***_H_**α = 20...40 G; dimeric radical cation [25], *g* = 2.010, *a***_H_**α = 9.5 G). The main difference between **DES** and **THTP** is a faster decay of the polarizations by the self-exchange in the case of the cyclic sulfide.

For the general reaction mechanism of electron-transfer-induced radical pair formation followed by exchange cancelation, the integrated rate law for the polarization *I*_rel_ of the regenerated donor as a function of the time *t* after the laser flash is biexponential [17,19],

[3]
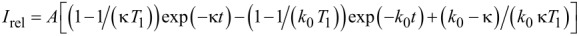


The fast component *k*_0_ in Equation 3 is the decay rate of the excited state whereas the slow component κ,

[4]
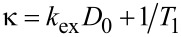


comprises the effects of self-exchange (rate constant, *k*_ex_; donor concentration, *D*_0_) and nuclear spin relaxation in the free radicals (relaxation time, *T*_1_). The latter spoils a perfect cancelation of geminate and escape polarizations, so is responsible for the residual magnetization at long times after the flash. Bimolecular recombination of the free radicals, which would lead to an additional term in Equation 4 [17], usually is of marginal importance only [19], and was neglected in our analysis because no dependence of κ on the laser intensity, and hence on the radical concentration, was observed.

Owing to technical reasons, NMR pulses often cannot be made infinitely short on the timescale of the polarization changes, and sampling time-dependent CIDNP with such ”long” pulses (duration, τ) then results in a convolution of pulse action and kinetics [42]. Under our experimental conditions, this convolution is tantamount to simply multiplying each exponential term exp(−*kt*) in Equation 3, where *k* is *k*_0_ or κ, with a constant factor,

[5]
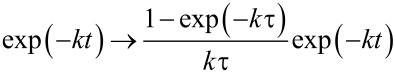


The changing of the weights of the two exponentials in Equation 3 by Equation 5 is the reason why the polarizations seen in Figure 1 do not start from zero at time zero after the laser flash.

**Figure 2:**
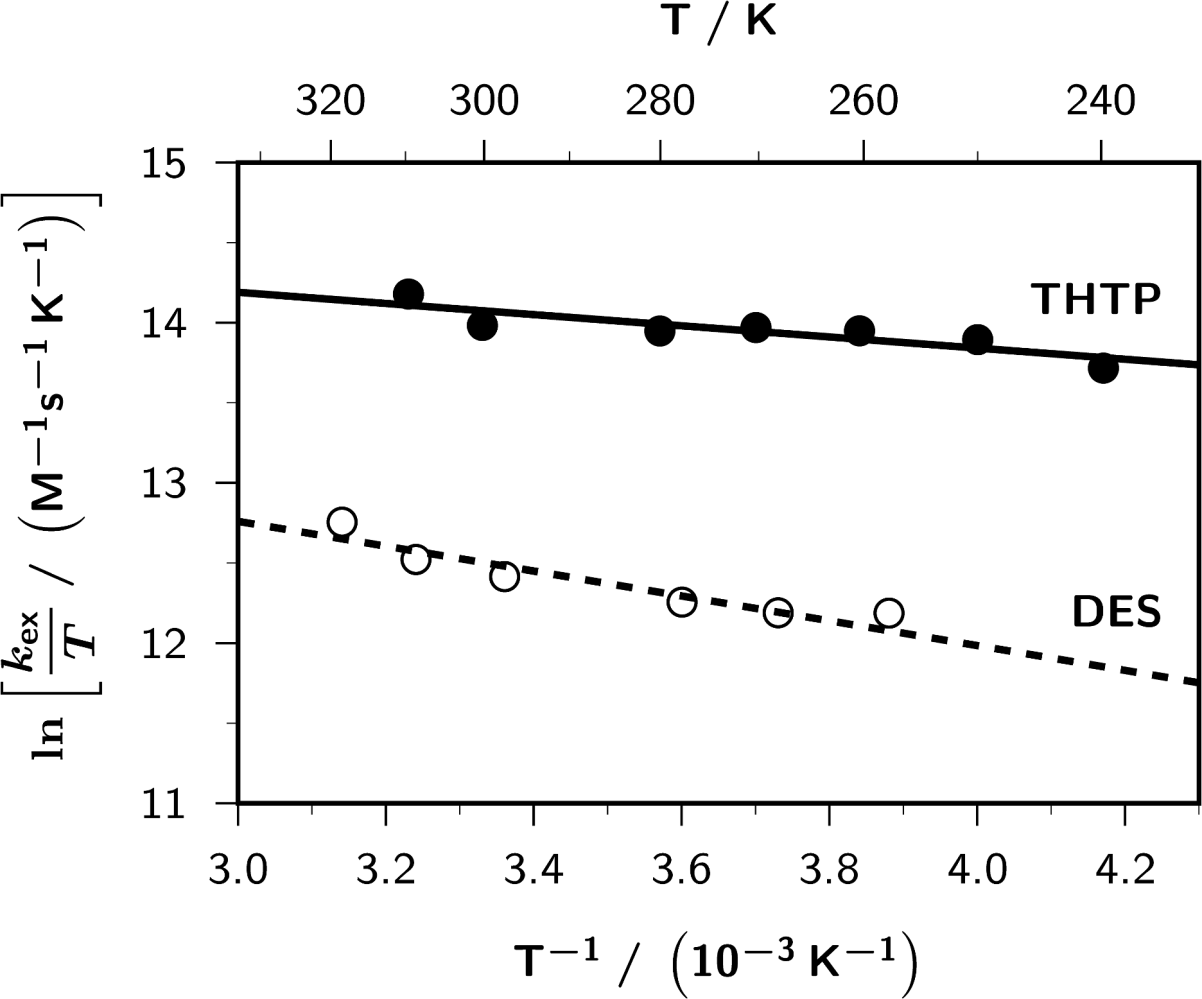
Eyring plots of the self-exchange rate constants *k*_ex_ for **DES** (open circles and broken line; linear regression, 15.08 – 775/*T*, *r*^2^ = 0.94) and **THTP** (filled circles and solid line; linear regression, 15.24 – 349/*T*, *r*^2^ = 0.88) in acetonitrile-*d*_3_; sensitizer **TTP**^+^. For all other experimental parameters and further explanation, see Figure 1 and text.

Eyring plots of the exchange rate constants so obtained for **DES** and **THTP** can be found in Figure 2, and Table 1 lists the activation parameters resulting from these plots. With **DES**, experiments at different concentrations gave slight systematic displacements of the regression lines, corresponding to about 20% lower apparent values of *k*_ex_ when the amount of sulfide was halved. We tentatively ascribe this to the dimerization of the radical cations discussed below; because of our high radical concentrations, the apparent depletion of **DES** by this complexation is higher for smaller substrate concentrations. On these grounds, we regard the intercept in Figure 2, and thus the activation entropy in Table 1, as a lower limit in the case of **DES**.

**Table 1:** Activation parameters (enthalpy 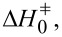 entropy 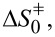 and free energy at room temperature 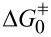 (298K)) for the self-exchange reactions of diethyl sulfide **DES** and tetrahydrothiophene **THTP** with their respective radical cations in acetonitrile.

Sulfide	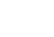 (kJ/mol)	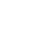 (J K^−1^ mol^−1^)	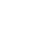 (298 K) (kJ/mol)

**DES**	6.4	−72.1	27.9
**THTP**	2.9	−70.9	24.0

The activation enthalpies are unusually small, but much more striking are the strongly negative activation entropies because it is well known that outer-sphere electron transfer of organic substrates in polar solvents is usually accompanied by small positive activation entropies [43] (e.g., our recent study on triisopropylamine [20] gave an activation entropy of +7.6 JK^−1^ mol^−1^). Our experimental values indicate transitions states that are much more ordered than usual, which is strong evidence that in these systems the self-exchange does not involve the monomeric radical cations **D^•^**^+^ but the dimeric ones 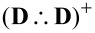, for which the formation of the new two–center three–electron bond greatly restricts the number of possible orientations of the reacting molecules.

Because of the considerable thermodynamic driving force [24], the initial formation of the dimeric cations must be much faster than the self-exchange, so cannot be captured by our time-resolved CIDNP experiments. It might even occur during the spin-correlated life of the radical pairs without being detectable by the polarization pattern because neither the *g*-value difference nor the distribution of unpaired spin density between the α and β protons changes in it, as discussed above.

Despite the obvious deviation of such an associative–dissociative process from a simple outer-sphere electron transfer, its experimental free energy of activation 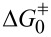 seems to be well reproduced by the Marcus theory in our case. That theory [1–2] predicts 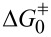 to be one quarter of the reorganization energy λ, which is the hypothetical energy needed for all the geometrical changes occurring in the reaction, but without actually transferring the electron. This includes converting the geometry of the nonradical species into that of the radical species, and vice versa (inner reorganization energy λ_i_), as well as rearranging the solvent shell to accommodate the charge distribution of the respective other side of the reaction equation (outer reorganization energy λ_0_).

In the majority of cases, λ_i_, which is usually calculated from the force constants and geometry differences of the reactants [1–2], constitutes but a small correction to the total reorganization energy, on the order of 10% [44]. At first glance, one would expect the situation to differ for our associative–dissociative electron transfers. For **DES**, however, all bond lengths and angles in the radical cation, regardless of monomeric or dimeric (except, of course, for the bond between the two sulfur atoms, which is only present in the dimer), and in the parent compound are extremely similar, with the maximum changes during the reaction lying well below 0.01 Å [45]; using the known valence force field for **DES** [46], one thus arrives at utterly negligible effects on λ_i_ from all these bonds. As regards the sulfur–sulfur bond, from the shape of its associated optical absorption band its potential energy curve was concluded [24] to be quite broad and shallow, so it seems reasonable to identify its contribution to λ_i_ with the free energy of the monomer–dimer equilibrium, which can in turn be estimated from the reported equilibrium constants [24] to lie around 20 kJ/mol. Because that is only about a fifth of the experimental total reorganization energy, the uncertainties of the approximation cannot play an important role; on the other hand, λ_0_ obviously also remains the dominant term by far, also for this reaction type.

The solvent dependence of 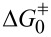 would allow a separation of λ_i_ and λ_0_, as only the latter is polarity dependent. Unfortunately, however, a variation of the solvent proved infeasible. In very nonpolar media, **TPP^+^** is not adequately soluble; in protic or nucleophilic solvents, the samples developed a brownish color after only a few laser shots and the CIDNP intensity decreased drastically, so time-resolved measurements could not be performed; in yet further solvents, such as DMSO, the residual signal of the solvent interfered with an exact determination of the polarization intensities, which are rather small in these systems owing to the large *g*-value differences.

Modeling the reacting molecules as spheres with diameters *a*_1_ and *a*_2_ and a distance *d* between them, Marcus [1–2] has derived an expression describing λ_0_ for the self-exchange between a neutral molecule and an ion of charge *z*,

[7]
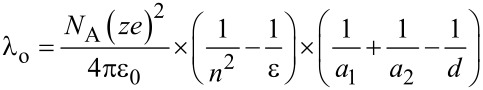


with *N_A_*, *e*, and ε_0_ being Avogadro’s number, the electron charge, and the vacuum permittivity. The second term of the product in Equation 7, the so-called polarity factor, characterizes the ability of the solvent to follow the charge redistribution during the exchange (*n*, refractive index; ε, relative permittivity); for acetonitrile, its value is 0.527 at room temperature. Several groups [47–48] including ours [49] have extended Equation 7 to multisphere models to take into account the charge distribution in the molecules.

In the associative–dissociative self-exchange between **D** and 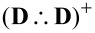, the charge distribution on one half of the dimer remains unchanged, so effectively *z* amounts to 1/2 only. Furthermore, there is no delocalization in our aliphatic substrates; hence, it seems natural to assume the exchanged charge to be basically localized on two of the three sulfur atoms. In this limit, the multisphere model again turns into Equation 7, with *a*_1_ and *a*_2_ being identical and representing the diameter of the charge cloud around each sulfur. There exists no clear criterion for deciding the value of *a*_1,2_, but choosing the length of the sulfur–sulfur bond in the dimer (2.9 Å [45]) as a reasonable approximation of this quantity and using the data of Table 1 as well as λ_i_ estimated above, one arrives at physically very reasonable distances *d* of 5.3 Å and 3.7 Å for **DES** and **THTP**. In the latter case, a closer approach is in line with expectation because the restricted geometric freedom and more compact configuration of the aliphatic substituents leave the sulfur atom more accessible; furthermore, the obtained reaction distance is practically equal to twice the van-der-Waals radius of sulfur [50], so is also very plausible.

## Conclusion

The present study has shed light on an unusual variant of electron self-exchange, namely, an associative–dissociative mechanism involving dimeric radical cations and monomeric parent compounds. It serves to illustrate the power of CIDNP to isolate certain steps within a complex reaction scheme and investigate their kinetics. Not only does it permit the identification of early, short-lived paramagnetic intermediates by freezing their spin-density distribution as a pattern of nuclear spin polarizations, but those polarizations then provide convenient labels that establish a connection with subsequent species on the reaction coordinate and, specific to this work, make the two sides of a self-exchange reaction distinguishable, as to allow one to measure its rate.

## Experimental

All chemicals were commercially available. **DES** was purified by distillation, **TPP**^+^ and **THTP** were used as received, the solvent acetonitrile-*d*_3_ was dried to a water content below 5 × 10^−4^ M in a dedicated apparatus [51]. Oxygen was removed by bubbling nitrogen through the ice-cooled (to reduce losses of sulfide and solvent) solutions; the samples were then sealed. The final concentration of the sulfide in the samples was determined by NMR spectroscopy. Time-resolved ^1^H (250 MHz) CIDNP measurements were performed on a Bruker WM 250 NMR spectrometer with a special probe [17] that provided illumination of the samples from the side. All unchanging background magnetization was removed by presaturation pulse sequences [52]. Excitation was done with a Lambda Physik EMG 101 excimer laser (medium, XeCl; wavelength, 308 nm; pulse width, 15 ns) triggered by the computer of the spectrometer. The probe was thermostated to ±0.3 K. Quantum mechanical calculations were carried out with the Gaussian 09 package [53] using the AM1 Hamiltonian.
